# Incidence of Postoperative Flare-Up After Single-Visit and Multi-visit Endodontic Therapy

**DOI:** 10.7759/cureus.57995

**Published:** 2024-04-10

**Authors:** Denitsa Zaneva-Hristova, Tsvetelina Borisova-Papancheva

**Affiliations:** 1 Department of Conservative Dentistry and Endodontics, The Faculty of Dental Medicine-Varna, Varna, BGR; 2 Department of Conservative Dentistry and Endodontics, Medical University of Varna, Varna, BGR

**Keywords:** multi-visit endodontic therapy, single-visit endodontic treatment, chronic apical periodontitis, flare-up, postoperative pain

## Abstract

Summary: This article presents results obtained from a survey, including patients who underwent endodontic treatment by the single-visit or multi-visit method, after confirmation of the diagnosis of chronic apical periodontitis.

Objective: The aim of the survey was to obtain data from the studied patients on the frequency and the type of postoperative pain after treatment of chronic apical periodontitis, as well as whether there is a relation between gender, age, and postoperative pain.

Materials and methods: A visual analog scale was used to study the intensity of postoperative pain in the treatment of teeth diagnosed with CPP, which are treated by one of two methods - single-visit or multi-visit method. The total number of surveyed patients is 71. The patients were examined and treated at the Dental Clinic "Imperial" in Varna, Bulgaria, in 2020. Thirty-one of them were treated by the single-visit method, and the remaining 40 by the multi-visit method with placement of a temporary dressing or sterile swab.

Results: A relatively large proportion (70%) of patients reported mild pain immediately after the root canal filling. A relatively large proportion (90.3%) of patients did not report pain one week after the root canal filling. The more frequent symptoms were observed in cases treated by the multi-visit method, after the application of a temporary dressing. Patients who reported taking analgesics were treated in the multi-visit method. More frequent pain symptoms with both methods of treatment were observed in men aged 36-60 years.

Conclusion: Although exacerbation has been shown to have no significant effect on the outcome of endodontic treatment, it is highly undesirable. In the short term, the postoperative pain in patients treated by the multi-visit method through the use of intracanal medication is more pronounced. Patients receiving the single-visit treatment reported less postoperative pain.

## Introduction

Postoperative pain is the most common complication at the end or between visits of endodontic treatment for teeth with chronic apical periodontitis, leading to patient discomfort, tension, and anxiety. Postoperative sensitivity is often underestimated. Due to the progress of medicine and the emergence of new techniques and materials, in our opinion, there is an opportunity to minimize it [1]. Therefore, the question of the effect of different treatment techniques and drugs used to reduce pain is debated in the literature. One of these techniques is laser therapy, which is useful and effective for everyday dental practice [2].

In one of the studies on this topic, it was found that antimicrobial photodynamic therapy leads to a reduction in postoperative pain after one-visit treatment and reduces the number of complications by almost 1.5 times, accelerating the process of recovery of the focus of bone destruction, which allows endodontic treatment to be performed in one visit [3]. Photodynamic therapy, used as an adjunct to conventional endodontic therapy, achieves a significant reduction of bacteria in the root canal system [4-6].

There are many unresolved issues related to postoperative sensitivity, its duration relative to the approach and medication used, and the relationship between preoperative and postoperative symptoms. Despite numerous studies related to temporary inlay medications, there are no confirmatory results related to their benefits in terms of microorganism reduction and postoperative pain.

Several studies have found greater postoperative sensitivity in teeth treated with a calcium hydroxide dressing [7-10]. Other authors reported that the temporary dressing did not help reduce postoperative pain [11,12].

However, in other studies, the authors found that complications, such as edema and analgesic intake, were more common in teeth with one-visit endodontic treatment.

On a global scale, the treatment of chronic periapical periodontitis by a one-visit method is increasingly being used.

A number of authors have compared the postoperative sensitivity after treatment of these teeth by the two methods - one-visit and multi-visit treatments. Such comparisons date back to 1982. In this study, the authors found that there was no significant difference in the incidence of pain in teeth treated with single-visit and multi-visit methods [13]. Similar results have been reached by other authors over the years [14-17].

Among Bulgarian dentists, this method of treating teeth with a diagnosis of chronic asymptomatic periapical periodontitis is still not accepted. For all the reasons listed, we find the unresolved issues relevant, which led to the formation of our goals and objectives published in the dissertation defended in 2021 by Dr. Denitsa Zaneva-Hristova at the Medical University-Varna.

After reviewing the literature, it is found that the advantages and disadvantages of the two treatment methods are examined on different indicators. This necessitates a thorough study regarding the medications used, their qualities, postoperative pain, and the healing process. After reviewing various articles on the topic of postoperative pain, in teeth with chronic apical periodontitis treated with both methods, no definitive results were obtained as to which method was used and which inlay resulted in less postoperative pain. We believe that this topic is relevant and important to dental practitioners and patients, which provoked the publication of our results confirming the results of other studies.

## Materials and methods

A certain number of male and female patients between the ages of 16 and 69, of Bulgarian origin, healthy, and without established systemic diseases, are required for the performance of the assigned tasks. Each person has one or more teeth on the upper, lower, or both jaws and is diagnosed with chronic apical periodontitis except for the wisdom teeth. The diagnosis was made based on clinical and paraclinical studies. The teeth are asymptomatic and do not respond to thermal tests. The values ​​reported from electroodontodiagnostics are above 100 µA. Radiographic diagnostics is one of the main methods used to verify the diagnosis of chronic apical periodontitis. Demineralization and destruction of periodontal tissues, cementum, and alveolar bone tissue are detected by periapical X-ray. Cone-beam computed tomography systems (CBCTs) can be used to confirm the diagnosis.

Contraindications for the inclusion of the teeth in the study can be general and local. The systematic criteria for excluding patients from the study are pregnancy, patients who had used antibiotics in the past month, and diabetics. The lack of sufficient structure and the impossibility of restoring it due to the presence of the ferrule effect for isolation with the help of a rubber dam is one of the most important contraindications for including the determination of teeth in this study.

There are several criteria for teeth with previous endodontic treatment to be included in the study. First, one should be able to fully negotiate the root canals. Second, the canal filling should reach the middle third of the canal leaving the apical part not filled. Lastly, the presence of separated instruments in the examined canals is inadmissible.

For the needs of the study, a special card was created, which was filled out by the patients. The patients were examined and treated at the Dental Clinic "Imperial" in Varna, Bulgaria, in 2020. The total number of examined patients was 71. The participants were divided according to three criteria. One is relative to the method of treatment. Thirty-one of them were treated using the single-visit method and 40 using the multi-visit method. The other two criteria are gender and age, dividing the sample into four age subgroups. The first includes people aged 16 to 18, and the second includes people aged 19 to 35. The third subgroup includes people from 36 to 60 years of age, and the last includes people over 60 years of age.

The patients treated by the one-visit method were 13 men and 18 women. There were no registered patients in the first and last age subgroups. In the second subgroup, there were six men and 10 women, and in the third subgroup (36-60 years), there were seven men and eight women.

The patients treated by the multi-visit method, divided by gender, were 18 men and 22 women. Again, the patients were divided into four subgroups according to their age. There were no registered patients in the first subgroup (16-18 years). In the second subgroup (19-35 years), there were eight men and 10 women. In the third subgroup (36-60 years), there were nine men and 10 women. The fourth subgroup included patients over 60 years of age: one man and two women.

The questionnaire includes a record of pain over a period of time after root canal obturation. The visual analog scale (VAS) [18] was used to monitor the level of pain during five different time intervals - immediately after the obturation of the root canals, in six hours, in 24 hours, in 48 hours, and seven days after filling them (Appendix A).

If the multi-visit method is used to treat chronic apical periodontitis, the data are also reported after the placement of the intracanal medication dressing. Some additional questions were included and appeared in the questionnaire about the type of pain, what provoked it, whether there was swelling during the treatment, whether the patient took analgesics, and how they affected the pain (Appendix B).

The aim of the survey is to gather data from the patients about the frequency and the type of postoperative pain after the treatment of chronic apical periodontitis and whether there is a correlation between gender, age, and postoperative pain. The analysis of the experimental data was performed with a specialized statistical analysis package IBM SPSS Statistics for Windows, version 20.0 (released 2011, IBM Corp., Armonk, NY). A p-value that is less than the chosen significance level α is chosen as the significance level. This is the probability of making a first-order error, that is, of detecting the null hypothesis when it is true. Descriptive statistics and statistical inferences were applied for search purposes.

## Results

Results from a survey of patients treated using the single-visit method

**Figure 1:**
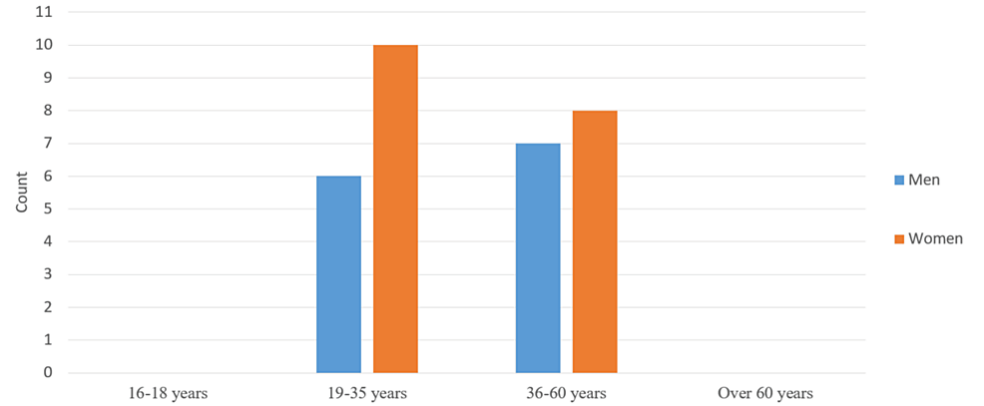
Distribution of patients treated using a one-visit method according to their age

We divided the patients into two groups according to their gender: men (13, 41.9%) and women (18, 58.1%). All patients were divided into four subgroups according to their age. No patients were registered in the first subgroup (16-18 years old). In the second subgroup (19-35 years old), there were six men and 10 women. In the third subgroup (36-60 years old), there were seven men and eight women. The fourth subgroup included patients over 60 years of age. There were no registered patients in this group (Figure 1).

**Figure 2:**
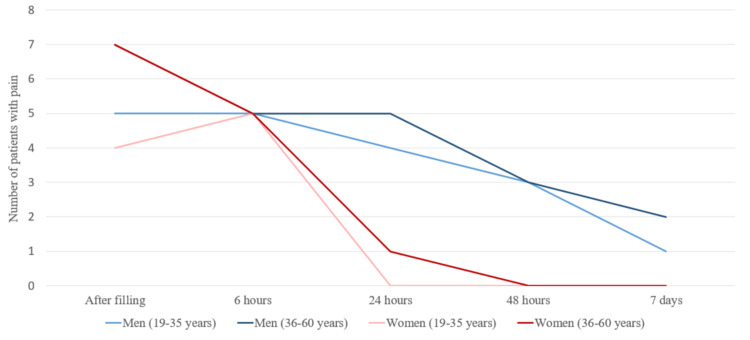
Reporting pain according to the gender and age of the patients

Based on the data collected from the patients, it has been found that in 12 male patients (92.3%), symptoms of pain and discomfort were present immediately after the obturation filling. Six patients (46.2%) reported persistent pain 48 hours after root canal filling. All were men, three from each of the two groups; 23.1% were 19-35 years old and 36-60 years old. Three patients (23.1%) reported pain one week after the treatment, as well as percussion pain. Only one female patient (5.6%) from the 36-60 years age group reported persistent mild pain 24 hours after root canal filling, which were absent during the next period of reporting (Figure 2).

**Figure 3:**
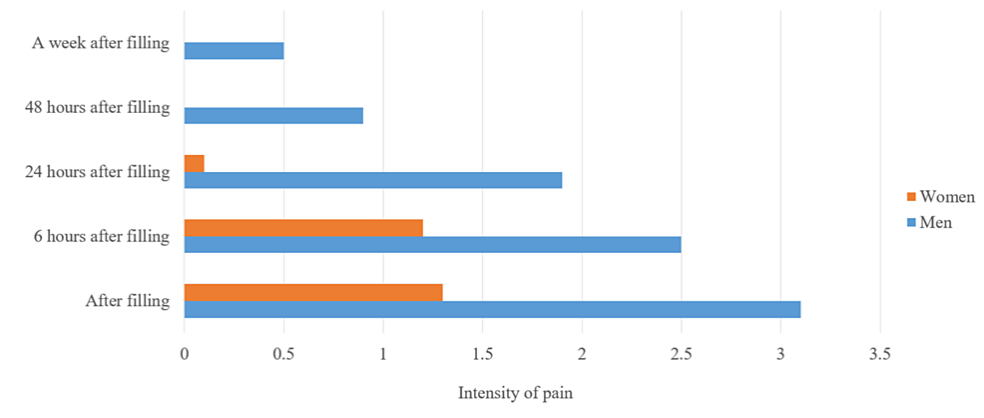
Average pain values in patients in different periods, divided according to their gender

We also took into account the average value of pain in men and women between the different periods of reporting (Figure 3).

**Figure 4:**
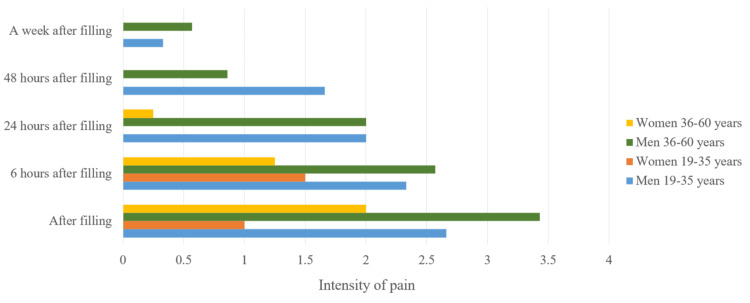
Average pain values in the patients

Pain symptoms in men at each stage of the treatment were more pronounced. We studied the pain symptoms in men and women in the different age groups. There were no registered patients in the 16-18 age group. After averaging the values, we have concluded that more frequent pain symptoms were present in men from both groups (Figure 4).

**Figure 5:**
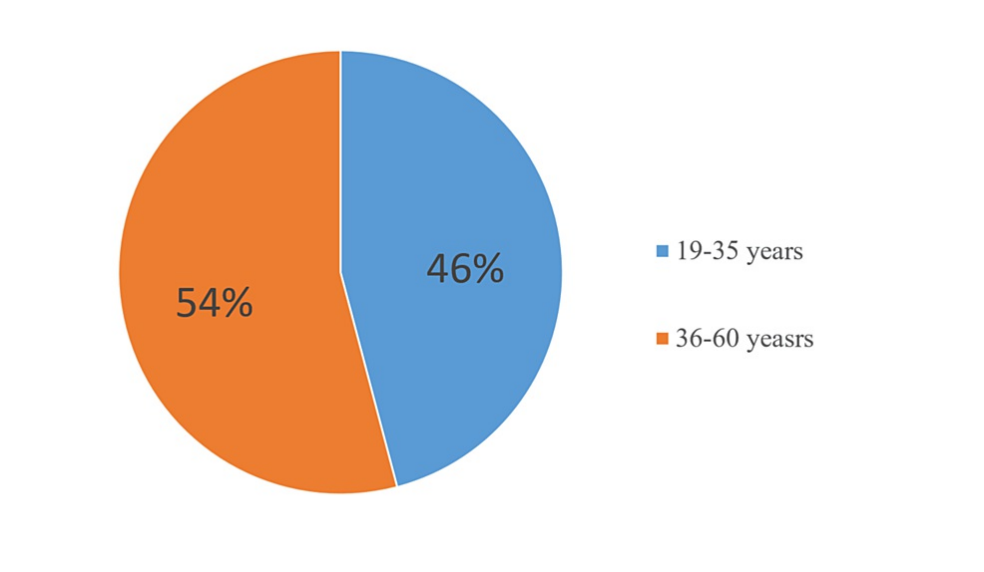
Average pain values in men from both age groups

**Figure 6:**
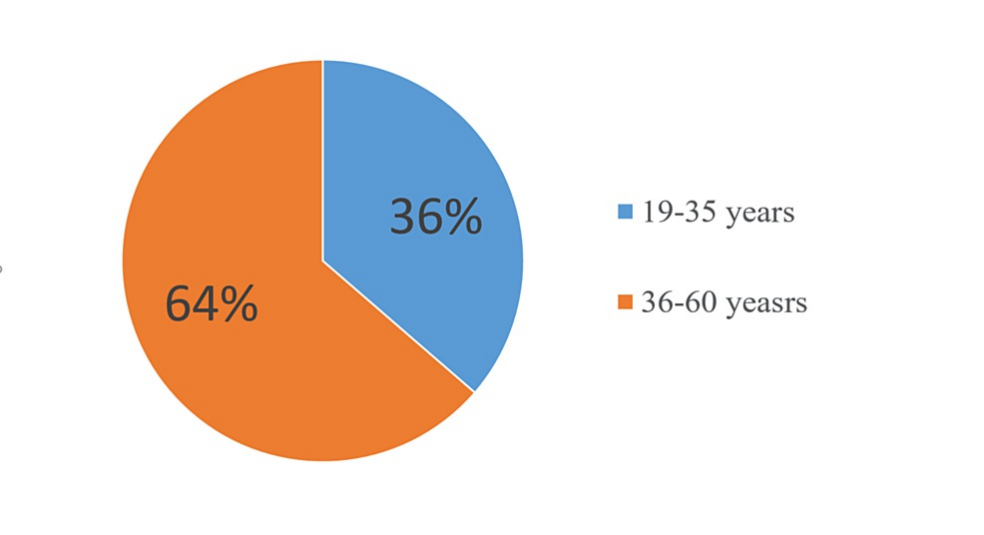
Average pain values in women from both age groups

We studied men and women separately and compared the data provided to us according to the different age groups (Figures 5, 6).

According to the results, a conclusion was made that patients aged 36-60 reported more pain on average compared to the 19-35 age group. The results have shown that women between the ages of 36 and 60 had more pronounced symptoms than the ones from the previous group.

Statistical analysis of the results obtained from a survey of patients treated using the single-visit method

The analysis of the experimental data was performed with the IBM SPSS Statistics for Windows, version 20.0 (released 2011, IBM Corp., Armonk, NY), which specializes in statistical analysis. A statistically significant difference between the results before and after the treatment was established.

Results from patients treated using the multi-visit method

**Figure 7:**
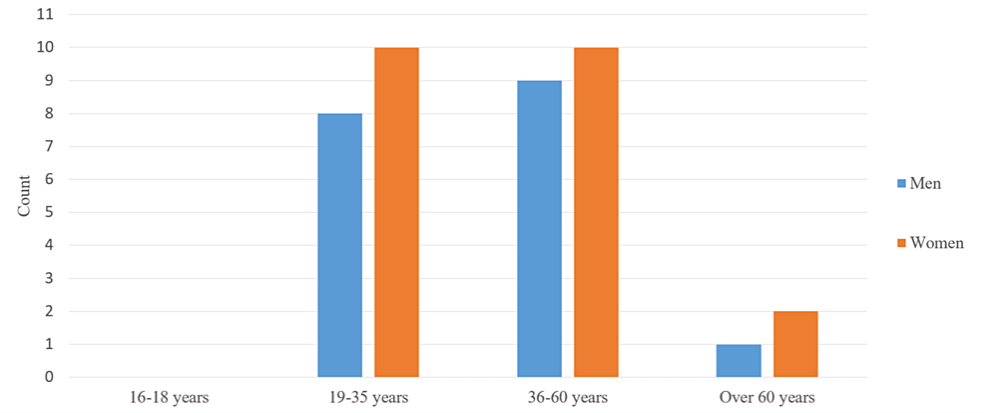
Distribution of patients, treated using the multi-visit method, by age

We divided the patients into two groups according to their gender: men (18, 45%) and women (22, 65%). All patients were divided into four subgroups according to their age (Figure 7).

**Figure 8:**
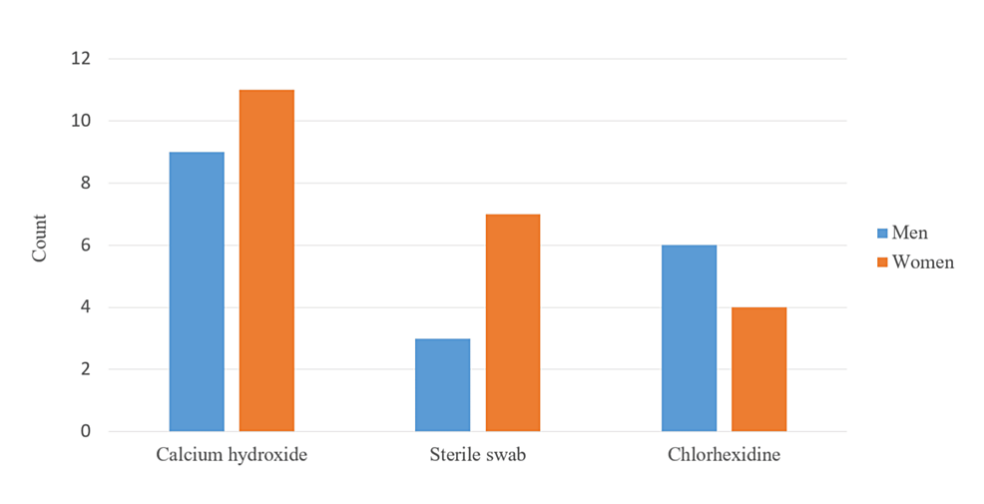
Distribution of patients by gender according to the medication applied

The surveyed 40 patients were divided into three groups according to the dressing used in their treatment and their gender. The first group included 20 people (50%) treated with a calcium hydroxide dressing. The second group was treated by applying a sterile cotton swab, and the third group was treated with 2% chlorhexidine for irrigation (Figure 8). Ten people were studied in the second and third groups.

Based on the data collected from the studied patients, 14 male patients (77.8%) reported symptoms during the first period. After filling the root canal, this percentage increased to 88.9%. These were two more patients from the 36-60 age group. Five patients (27.8%) reported persistent pain seven days after root canal filling. Four of them were in the 36-60 age group and one in the 19-35 age group.

**Figure 9:**
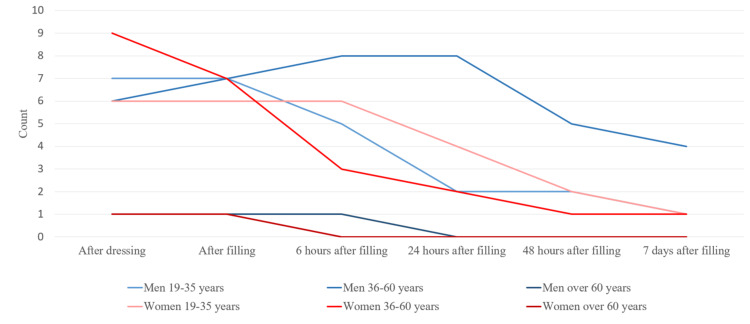
Intensity of pain according to the gender and age of the patients

Based on the data collected from the studied patients, 16 female patients (72.8%) reported symptoms, which included pain and discomfort during the first period. After filling the root canal, this percentage decreased to 63.6%. Only one female patient from the 19-35 age group and one from the 36-60 age group reported persistent mild pain seven days after root canal filling (Figure 9). A total of three people (7.5%) reported severe pain - six (from VAS) during all reporting periods. All of them were male patients from the 36-60 age group.

**Figure 10:**
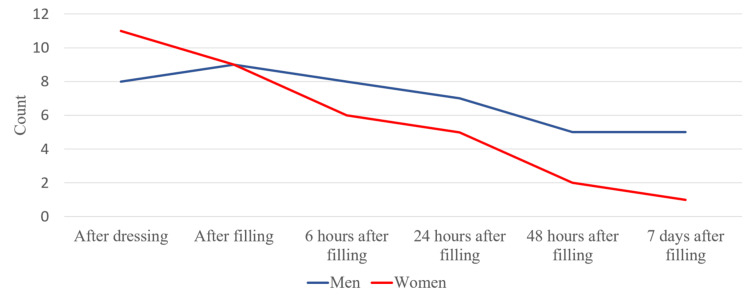
Intensity of pain in the multi-visit treatment after the application of calcium hydroxidе

After analyzing the data obtained from the patients, we found that all the women (100%) treated with calcium hydroxide dressing reported pain after the application of the medicaments. Eight men (88.9%) reported pain after the first stage of the survey. In the next stage of the study, all men (100%) reported the presence of symptoms, and these values stayed the same in the next stage of the study. Nine women (81.8%) reported pain immediately after root canal filling. The data show that seven days after the treatment, five men (55.6%) and one woman experienced persisting pain (Figure 10).

**Figure 11:**
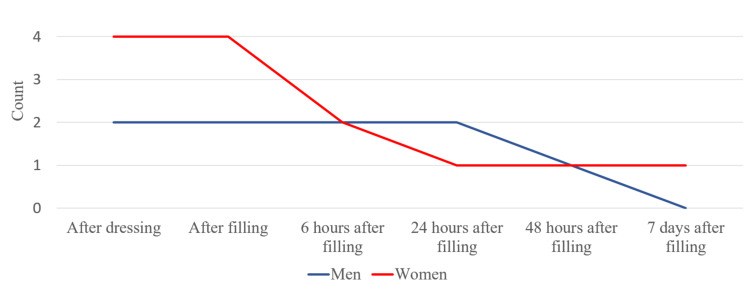
Intensity of pain in the multi-visit treatment without a dressing

After analyzing the data obtained from the patients, we found the same values in the first two stages of the survey - after placing a dry sterile swab and immediately after filling the root canal. Two of the surveyed men (66.6%) and four of the women (66.6%) reported pain. The data show that in one woman (14.28%), pain persisted seven days after the treatment (Figure 11).

**Figure 12:**
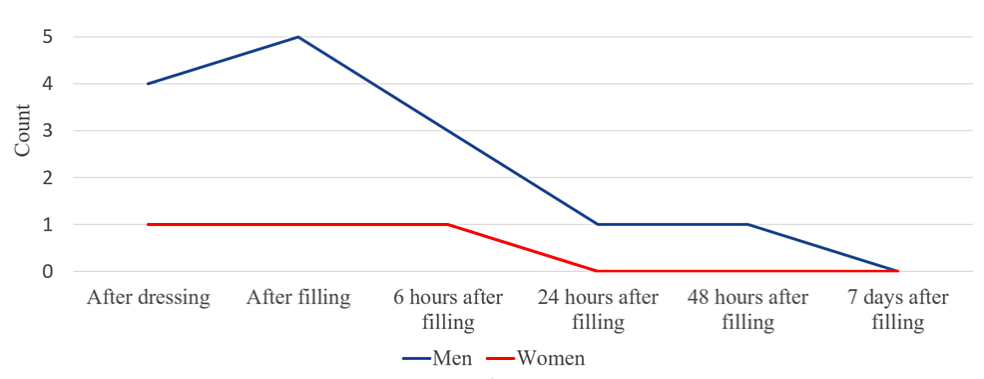
Intensity of pain in the multi-visit treatment after chlorhexidine (CHX)

After analyzing the data obtained from the patients, we found that one woman (25%) out of the patients treated with chlorhexidine reported pain after it had been applied. Four men (66.7%) reported pain after the first stage of the survey. In the next stage of the study, the number of men with symptoms increased to five (83.3%), and these values ​​decreased in the following survey periods. Only one woman (25%) reported pain immediately after the root canal filling. The data show that none of the surveyed men and women reported pain and discomfort seven days after treatment (Figure 12).

**Figure 13:**
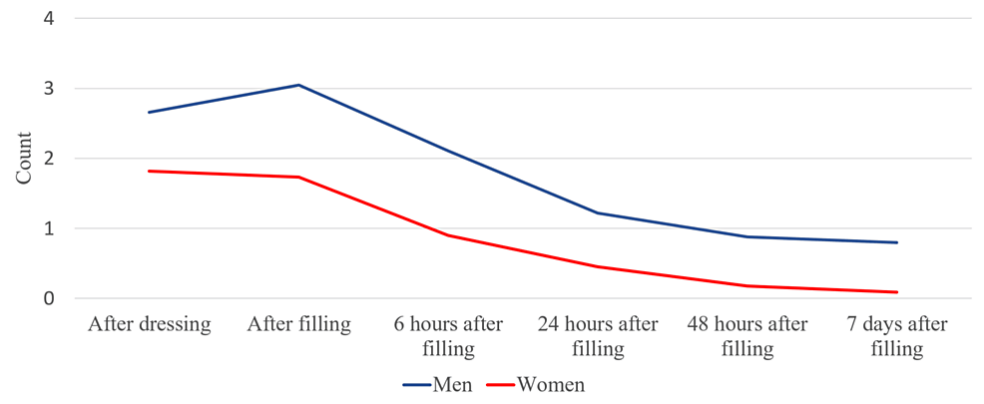
Average pain values in different periods in patients divided by their gender

We took into account the average pain values in men and women and between the different periods of reporting pain, as shown in Figure 13.

At each stage of their treatment, we observed that men experienced more pronounced pain.

**Figure 14:**
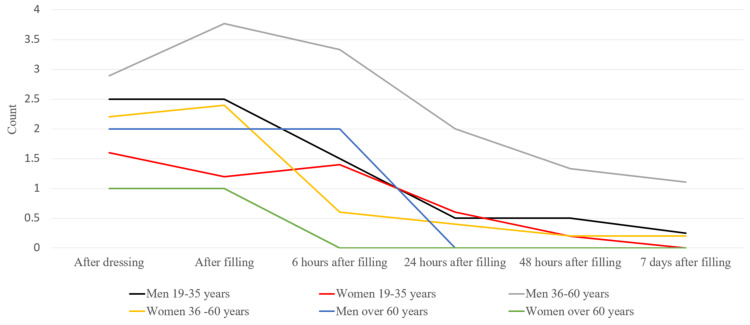
Average pain value in the patients

We examined the pain symptoms in men and women from the different age groups. There were no patients in the 16-18 age group. After averaging the values, we found that overall men had more frequent pain symptoms no matter the age group (Figure 14).

**Figure 15:**
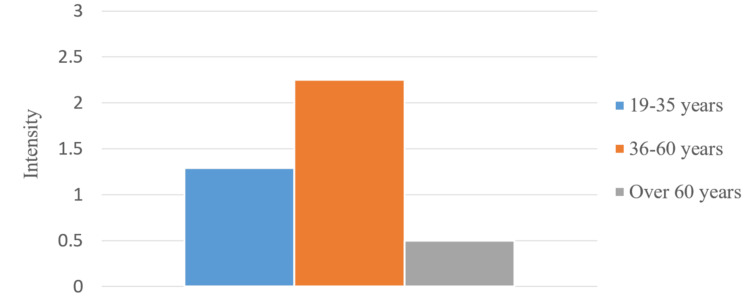
Average pain values in men from different age groups

**Figure 16:**
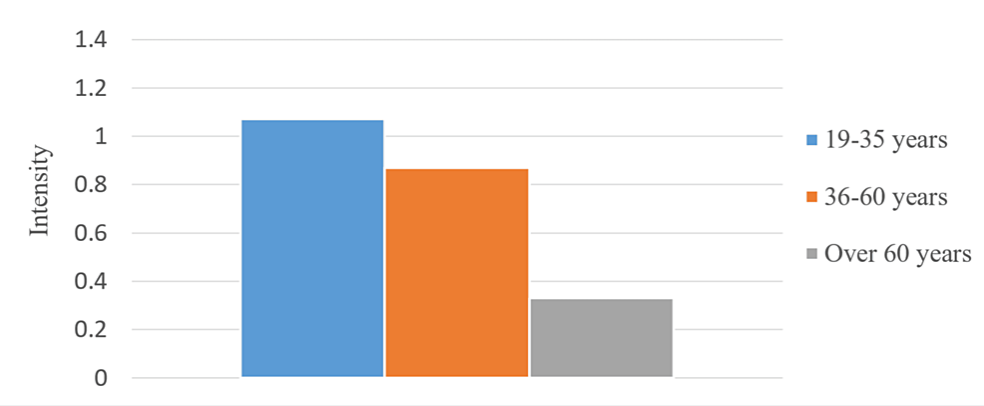
Average pain values in women from different age groups

We studied men and women separately and compared the data from the different age groups. We calculated the average pain values in regard to its intensity for the total survey period (Figures 15, 16). In the patients from the middle age group, there were more pronounced symptoms compared to the other two groups. Patients between 19 and 35 years had more pronounced symptoms compared to the other two groups. 

## Discussion

Discussion of the results obtained from the survey of the patients who were treated by a one-visit method

Based on the data obtained from the surveyed patients, it has become clear that men had more pronounced symptoms during different periods of the study, which differs from the results obtained by other authors [19].

When dividing patients by age groups, we have found that those in the 36-60 age group were more likely to report pain symptoms, which may be related to changes in humoral and cell-mediated immunity due to aging.

Based on the statistical analysis, we have found a statistically significant difference (p-value = 0.0000) between the first period of pain reporting (immediately after filling the root canal) and the remaining reporting periods. The reasons for this may be due to the shorter exposure to irrigation solutions and medications that may induce an immune response in single-visit treatment methods, as other authors have also confirmed [20,21].

Discussion of the results from patients treated using the multi-visit method

The present study has demonstrated that 100% of the women treated with a calcium hydroxide dressing experienced pain after the medication was applied. This may be due to the tendency to develop postoperative pain as they are more prone to psychosomatic disorders [22,23]. Another study explains the more pronounced symptoms in women with variable levels of the hormones serotonin and estrogen [24-26].

Changes in female hormones during menstruation, hormone replacement therapy, and taking oral contraceptives may alter serotonin and norepinephrine levels, thus contributing to a reduced pain threshold [27].

It was noted that a larger percentage of the patients reported persisting symptoms following multiple visits after treatment. This is likely due to the longer exposure to irrigants and medications, resulting in an immune response after irritation of the periapical space.

When dividing patients into groups according to their age, we have found out that the male patients aged 36-60 are more likely to report pain symptoms, which may be related to changes in humoral and cell-mediated immunity with aging [19]. These results are similar to the study of pain in patients treated using the single-visit method.

Results obtained from the survey of patients with chronic periapical periodontitis to whom one of the two studied treatment methods was used

A relatively large proportion (70%) of patients treated by both methods reported mild pain immediately after filling the root canal, which was also confirmed in studies by other authors [28]. The majority of patients (67.7%) reported no pain 24 hours after root canal filling. A relatively large proportion (90.3%) of patients reported no pain one week after root canal filling, which overlaps with the results of another study [29].

The more pronounced symptomatology is observed in cases treated by the multi-visit method, after the application of a temporary dressing [9]. This was also confirmed by the study of Hepsenoglu et al. [30]. Patients who received analgesics were treated in the multi-visit method.

After the end of the study, some limitations were observed. The patients were treated in one medical facility. The study period of the patients was short, due to additional tasks concerning long-term follow-up of the healing process of the teeth included in the study. Patients who did not return for follow-up radiographs were excluded from the study. Due to the facts listed above, the number of patients is not sufficient to create a representative sample, which leads to inconclusive results. Further research is needed on postoperative sensitivity in teeth with chronic apical periodontitis.

## Conclusions

We found that patients treated with the multi-visit method reported more frequent persistent symptoms during and after treatment compared with data obtained from patients treated with the single-visit method. This may be due to the fact that they were exposed to medication and irrigants for a longer period of time, which resulted in an immune response due to irritation of the periapical space. After reviewing all the facts presented, we recommend using the one-visit endodontic treatment. From a statistical point of view, we have no reason to give preference to either of the two CPP treatment techniques (p-value > 0.05).

## Figures and Tables

**Figure 17:**
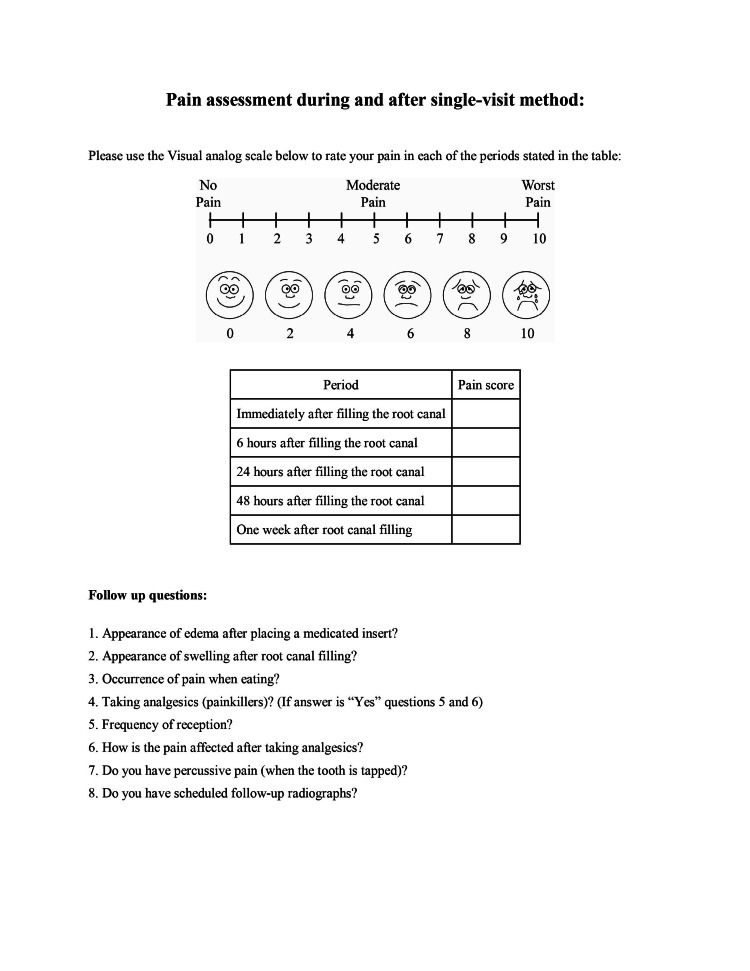
Questionnaire for patients treated using the single-visit method

**Figure 18:**
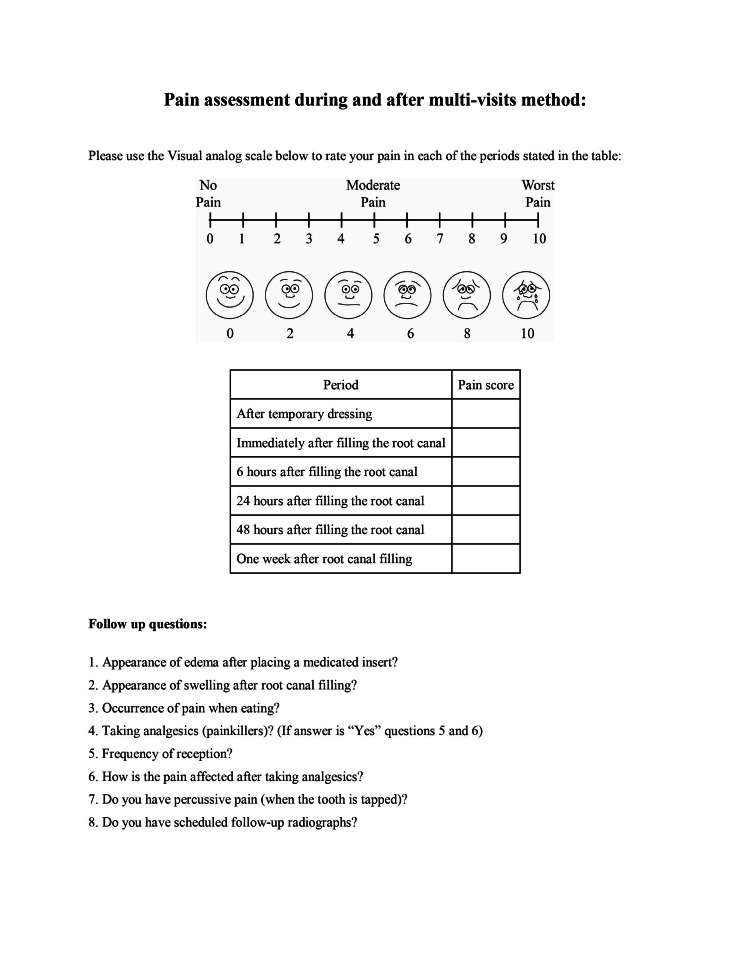
Questionnaire for patients treated using the multi-visit method

## Appendices

Appendix A

Appendix B

## References

[1] AlRahabi MK (2017). Predictors, prevention, and management of postoperative pain associated with nonsurgical root canal treatment: a systematic review. J Taibah Univ Med Sci.

[2] Aldelaimi TN, Khalil AA (2015). Clinical application of diode laser (980 nm) in maxillofacial surgical procedures. J Craniofac Surg.

[3] Karakov KG, Gandylyan KS, Khachaturyan EE, Vlasova TN, Oganyan AV, Eremenko AV (2018). Comparative characteristics of the methods of treatment of chronic periodontitis using antibacterial photodynamic therapy (per one visit) and calasept preparation. J Natl Med Assoc.

[4] Arslan H, Doğanay E, Karataş E, Ünlü MA, Ahmed HM (2017). Effect of low-level laser therapy on postoperative pain after root canal retreatment: a preliminary placebo-controlled, triple-blind, randomized clinical trial. J Endod.

[5] Jurič IB, Plečko V, Pandurić DG, Anić I (2014). The antimicrobial effectiveness of photodynamic therapy used as an addition to the conventional endodontic re-treatment: a clinical study. Photodiagnosis Photodyn Ther.

[6] Genc Sen O, Kaya M (2019). Effect of root canal disinfection with a diode laser on postoperative pain after endodontic retreatment. Photobiomodul Photomed Laser Surg.

[7] Ehrmann EH, Messer HH, Adams GG (2003). The relationship of intracanal medicaments to postoperative pain in endodontics. Int Endod J.

[8] Patil AA, Joshi SB, Bhagwat SV, Patil SA (2016). Incidence of postoperative pain after single visit and two visit root canal therapy: a randomized controlled trial. J Clin Diagn Res.

[9] Yousaf O, Khan K, Naz F (2016). Postoperative pain comparison in single versus two visit endodontics treatment. Pak Oral Dental J.

[10] Almeida DO, Chaves SC, Souza RA, Soares FF (2017). Outcome of single- vs multiple-visit endodontic therapy of nonvital teeth: a meta-analysis. J Contemp Dent Pract.

[11] AbdurRahman S, Abdel Aziz SM, Gawdat SI, AbdalSamad AM (2019). Postoperative pain of patients with necrotic teeth with apical periodontitis following single visit endodontic treatment versus multiple visit endodontic treatment using triple antibiotic paste: a randomized clinical trial. F1000Res.

[12] Tarale K (2013). Post-operative pain analysis between single visit and two visit root canal treatments using visual analogue scale: an in vivo study. J Dent Allied Sci.

[13] Mulhern J, B Dent, Patterson S, Newton C, Ringel A (1982). Incidence of postoperative pain after one-appointment endodontic treatment of asymptomatic pulpal necrosis in single-rooted teeth. J Endod.

[14] Manfredi M, Figini L, Gagliani M, Lodi G (2016). Single versus multiple visits for endodontic treatment of permanent teeth. Cochrane Database Syst Rev.

[15] Nunes GP, Delbem AC, Gomes JM, Lemos CA, Pellizzer EP (2021). Postoperative pain in endodontic retreatment of one visit versus multiple visits: a systematic review and meta-analysis of randomized controlled trials. Clin Oral Investig.

[16] Sheikh H, Fawzy M, Khalefa M, Bastawy H (2017). Clinical evaluation of single versus multiple visits endodontic retreatment outcome: a randomized controlled trial. Al-Azhar J Dent.

[17] Wong AW, Zhang C, Chu CH (2014). A systematic review of nonsurgical single-visit versus multiple-visit endodontic treatment. Clin Cosmet Investig Dent.

[18] Hayes M, Patterson D (1921). Experimental development of the graphic rating method. Psychol Bull.

[19] Nair M, Rahul J, Devadathan A, Mathew J (2017). Incidence of endodontic flare-ups and its related factors: a retrospective study. J Int Soc Prev Community Dent.

[20] Su Y, Wang C, Ye L (2011). Healing rate and post-obturation pain of single- versus multiple-visit endodontic treatment for infected root canals: a systematic review. J Endod.

[21] Kalhoro FA, Mirza AJ (2009). A study of flare-ups following single-visit root canal treatment in endodontic patients. J Coll Physicians Surg Pak.

[22] Ng YL, Glennon JP, Setchell DJ, Gulabivala K (2004). Prevalence of and factors affecting post-obturation pain in patients undergoing root canal treatment. Int Endod J.

[23] Colameco S, Becker LA, Simpson M (1983). Sex bias in the assessment of patient complaints. J Fam Pract.

[24] Marcus D (1995). Interrelationships of neuro-chemicals,estrogen, and recurring headache. Pain.

[25] Dao TTT, Knight K, Ton-That V (1998). Modulation of myofascial pain patterns by oral contraceptives: a preliminary reports. J Prosthet Dent.

[26] Mathew S (2015). Post operative pain in endodontics: a systemic review. J Dent Oral Hyg.

[27] Fillingim RB, Maixner W (1995). Gender differences in the responses to noxious stimuli. Pain Forum.

[28] Jabeen S, Khurshiduzzaman M (2013). A study of post obturation pain following single visit root canal treatment. Chattagram Maa-O-Shishu Hosp Med College J.

[29] Wong AW, Zhang S, Li SK, Zhu X, Zhang C, Chu CH (2015). Incidence of post-obturation pain after single-visit versus multiple-visit non-surgical endodontic treatments. BMC Oral Health.

[30] Erdem Hepsenoglu Y, Eyuboglu TF, Özcan M (2018). Postoperative pain intensity after single- versus two-visit nonsurgical endodontic retreatment: a randomized clinical trial. J Endod.

